# Characteristics of evolving models of care for arthritis: A key informant study

**DOI:** 10.1186/1472-6963-8-147

**Published:** 2008-07-14

**Authors:** Crystal MacKay, Paula Veinot, Elizabeth M Badley

**Affiliations:** 1 Arthritis Community Research and Evaluation Unit (ACREU), Toronto Western Research Institute, Toronto, Ontario, Canada; 2 Department of Physical Therapy, University of Toronto, Toronto, Ontario, Canada; 3 Department of Public Health Sciences and Graduate Department of Rehabilitation Sciences, University of Toronto, Toronto, Ontario, Canada

## Abstract

**Background:**

The burden of arthritis is increasing in the face of diminishing health human resources to deliver care. In response, innovative models of care delivery are developing to facilitate access to quality care. Most models have developed in response to local needs with limited evaluation. The primary objective of this study is to a) examine the range of models of care that deliver specialist services using a medical/surgical specialist and at least one other health care provider and b) document the strengths and challenges of the identified models. A secondary objective is to identify key elements of best practice models of care for arthritis.

**Methods:**

Semi-structured interviews were conducted with a sample of key informants with expertise in arthritis from jurisdictions with primarily publicly-funded health care systems. Qualitative data were analyzed using a constant comparative approach to identify common types of models of care, strengths and challenges of models, and key components of arthritis care.

**Results:**

Seventy-four key informants were interviewed from six countries. Five main types of models of care emerged. 1) Specialized arthritis programs deliver comprehensive, multidisciplinary team care for arthritis. Two models were identified using health care providers (e.g. nurses or physiotherapists) in expanded clinical roles: 2) triage of patients with musculoskeletal conditions to the appropriate services including specialists; and 3) ongoing management in collaboration with a specialist. Two models promoting rural access were 4) rural consultation support and 5) telemedicine. Key informants described important components of models of care including knowledgeable health professionals and patients.

**Conclusion:**

A range of models of care for arthritis have been developed. This classification can be used as a framework for discussing care delivery. Areas for development include integration of care across the continuum, including primary care.

## Background

The burden of chronic disease is increasing with significant direct and indirect health costs to society.[1,2] As health care systems strive to cope with this increased burden of disease, the management of chronic conditions has been criticized for being inadequate [3] and often failing to meet the needs of people with chronic conditions by focusing on acute illness.[4,5] Proponents of improved chronic disease management advocate for health system changes to improve the quality of care.[5] While chronic care models have been developed to re-design health care services across the system for people with chronic disease, [4-8] more frequently models of care have been developed in an ad hoc manner to meet local needs.

Arthritis is a prime example of a prevalent chronic condition with concomitant barriers to accessing quality care. Arthritis is one of the most prevalent chronic conditions and the most frequent cause of physical disability in the adult population. [9-12] The burden of arthritis is projected to increase with the aging of the population. [11-13] People with arthritis experience pain, disability, reduction in societal participation including loss of employment, and altered quality of life.[10,12] Timely access to appropriate health care and related services is crucial to minimizing this impact.

There are increasing pressures in the health care system that impede delivery of care to this population.[12,14-18] Studies have documented deficiencies in the primary care management of arthritis including lack of appropriate referral for non-pharmacologic and specialist care as well as general practitioners' lack of confidence in musculoskeletal examination. [19-24] People with arthritis have identified not receiving needed rehabilitation services.[25] There are shortages and geographic variation in availability of specialists, including rheumatologists and orthopaedic surgeons.[12,15,18] Persistent long waiting times have been documented, particularly for total joint replacement.[26] Delayed access to total joint replacement has been shown to lead to worse functional outcomes.[27] In the case of inflammatory arthritis, long waits may prevent patients from accessing medications that can slow disease progression and which are most effective in the very early stages of the disease.[28,29]

Evidence-based care points to the need for management of arthritis as a chronic disease with interventions directed to the reduction of pain, and prevention of disability as well as timely and appropriate access to specialist care. The challenge to providing timely and comprehensive quality care is a common international problem that has elicited the development of a number of alternative approaches to care delivery for arthritis. Health care providers such as physiotherapists, occupational therapists and nurses play a key role in many of these programs. Prior research has focused on the development and evaluation of specific programs or services. The use of teams to manage arthritis care is a long-standing tradition in some countries and has been shown to be effective in improving patient outcomes. [30-33] There is a growing body of literature addressing the use of health care providers, mainly physiotherapists and nurses, in expanded roles in rheumatology and orthopaedics to improve access to quality care. [34-43] However, most models of care have developed in response to local needs with limited evaluation.

The ad hoc manner of development of models of care and their limited evaluation pose challenges for their wider implementation. In addition, variability in the use of terminology to describe the models of care poses challenges to communication about service delivery. As the burden of arthritis increases in the face of shortages of health care providers and misdistribution of services, there is a growing recognition for the need for improved models of care. In order to facilitate development of models of service delivery that ensure quality and timely care, it is important to look at the breadth of models currently in use and the context in which they are employed. Despite the increased interest in exploring models of care for arthritis, there is a dearth of literature examining the range of types of models of care in different settings. The primary objective of this study is to a) examine the range of models of care that deliver specialist services using a medical/surgical specialist and at least one other health care provider and b) document the strengths and challenges of the identified models. As such, this paper focuses more on specialist models of care for individuals with inflammatory arthritis and more advanced osteoarthritis. A secondary objective is to identify key elements of best practice models of care for arthritis.

## Methods

Semi-structured interviews were conducted with a purposive sample of key informants who represented various models of care and/or were known as opinion leaders in arthritis care from primarily publicly-funded health care systems. A model of care was defined as a formalized approach or method for delivering care to individuals with arthritis. For the purposes of this paper, only models of care with provision of specialist services using medical/surgical specialists and other health care providers (e.g. physiotherapists, nurses) will be described. Models of care can also encompass services and programs delivered by non-health care providers within the community, an important often under-recognized component of care.

Key informants were selected for broad representation of professions, practice sectors and geographic variation. Key informants were identified using a snowball technique whereby the key informants who were interviewed were asked to recommend other individuals who could inform this study.

Participants were recruited between November 2004 and February 2006. Potential participants were contacted by telephone or electronic mail to enquire about their interest in participating in the study. Interested individuals were sent an information letter and consent form and subsequently contacted by a research associate to answer questions and arrange an interview time. Written consent was obtained prior to each interview.

Interview guides were developed based on a literature review of best practices in arthritis management and models of care for arthritis, as well as consultation with colleagues. The interview included mainly open-ended questions about structures and processes in the model of care, strengths and challenges, and key components of arthritis care delivery. Interview questions with sample probes are presented in Appendix 1. Interviews were primarily conducted by telephone (n = 59) and were audio-taped. Most interviews lasted less than one hour and the average was about 45 minutes. It was our intent to sample a wide range of participants representing a variety of different models of care. While it is difficult to determine saturation in a study of this scope, we considered saturation the point when no new types of models of care were identified.

### Analysis

Data were analyzed using a constant comparative approach.[44] A coding scheme was developed using an iterative, inductive approach. Initial open coding of the interviews was conducted by two research associates. Once a preliminary coding scheme was developed, two research associates independently used the coding scheme to code the interview data. They subsequently met to compare and contrast the use of codes until a satisfactory level of agreement was reached to develop the final coding scheme. The research associates coded each interview independently using this coding scheme. Following this, they met to review the coding to ensure consistency in the definitions and interpretations of codes. The coded data were entered into NUD*IST Version 6 (N6), a data software program designed to assist with management of qualitative data. The data were then examined to identify common themes across interviews.

Programs and services were systematically classified or categorized into types of models of care with similar characteristics and commonalities. The factors that were considered for classification were: 1) the method of delivery of care including who delivers the care (how); 2) the patient population (who); 3) types of interventions (what); 4) the location of care delivery (where); and 5) the rationale for the model of care (why) (e.g. comprehensive care or improving access to care). Although all factors were considered, the first factor, method of care delivery, was prioritized for assigning a primary designation of the program to a specific type of model of care.

This study was approved by the University Health Network Research Ethics Board.

## Results

### Sample Characteristics

In total, 74 key informants were interviewed (Table 1). Throughout the recruitment process, four individuals declined to participate in the study due to lack of time or information in the topic area. Most participants were from Canada (n = 59) while fifteen participants were from other countries. Fifty-eight participants worked primarily in clinical or administrative roles in health care delivery. This included managers and directors of clinical units. Sixteen participants worked primarily as researchers and academics, including academic leadership positions such as directors of academic units, professors and deans. They often held clinical and administrative appointments as well. The largest numbers of key informants were physiotherapists (25), nurses (12), and rheumatologists (11). On average, participants had 15.4 years experience working in the arthritis field, with a range of one to 40 years.

**Table 1:** Characteristics of Key Informants

**Key Informants**	**Number (74)**
**Country of Origin**	

Canada	59
Australia	1
Netherlands	2
Norway	2
Sweden	4
United Kingdom	6

**Profession**	

Physiotherapist	25
Rheumatologist	11
Occupational Therapist	6
Primary Care Physician	2
Psychologist	1
Nurse	12
Orthopaedic Surgeon	5
Social Worker	2
Other	10

Only data from key informants who described programs and services that met our inclusion criteria (include a medical/surgical specialist and at least one other health care provider) were included in the analysis for objective one. However, the perspectives of all key informants were included for analysis of the secondary objective, which aimed to identify key elements of arthritis care (n = 74).

### Types and Characteristics of Specialist Models of Care

**Figure 1:**
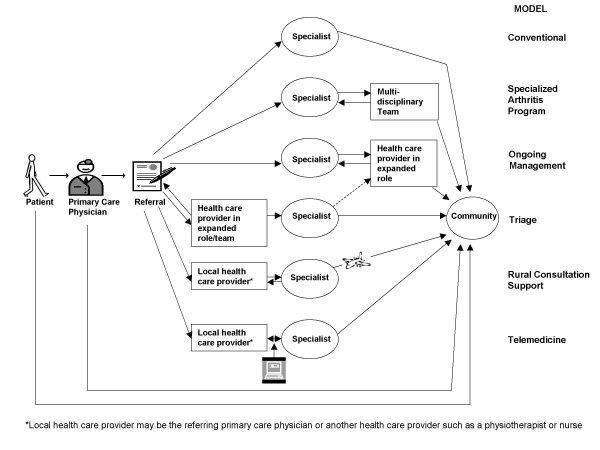
Types of Arthritis Models of Care.

The common models of care that emerged from our key informant interviews are illustrated schematically in Figure 1. We also include for comparison purposes the conventional model whereby the person with arthritis sees his/her primary care physician, who may then refer to an arthritis specialist such as a rheumatologist or orthopaedic surgeon.

#### Specialized Arthritis Programs

Programs and services that typify specialized arthritis programs are characterized by five key features: 1) services are primarily provided to individuals who have a diagnosis of arthritis; 2) a multidisciplinary team delivers care; 3) a broad range of interventions are available; 4) services are commonly provided in a hospital setting, including rheumatology departments and designated arthritis programs; and 5) the aim of the model is to provide comprehensive care.

Most commonly these programs focus on inflammatory arthritis patients with complex needs, such as rheumatoid arthritis, psoriatic arthritis and ankylosing spondylitis. Patients are typically referred by a primary care physician to a specialist, often a rheumatologist, for assessment and diagnosis. The patient is subsequently referred to other members of the health care team. Less frequently, patients are referred directly to an arthritis program for a team assessment. This model is characterized by the comprehensiveness of a range of services, including both individual and group interventions. Services include medical management, physiotherapy and exercise, occupational therapy and joint protection, and social work to deal with issues of stress, coping and finances. Patient education is a predominant feature of such programs in both group and individual formats.

The most common team members reported by key informants were rheumatologists, physiotherapists, occupational therapists, nurses, and social workers. Less frequently, dieticians, pharmacists, psychologists and orthopaedic surgeons were included as team members. Team communication and function were identified as key components of this model, including regular patient rounds or team meetings to discuss patient care and goal setting.

Two types of models of care involve the use of health care providers, most often nurses, physiotherapists and occupational therapists, in expanded clinical roles. The first model of care, the Ongoing Management model, uses health care providers in expanded roles to provide ongoing management of patients with arthritis working in collaboration with a specialist. The second, the Triage model, aims to assess patients with musculoskeletal conditions and refer to appropriate services. We considered expanded roles of practice as those requiring advanced clinical skills and knowledge, which often include performing additional acts such as ordering diagnostic tests or prescribing medications. The titles and expectations for these roles vary internationally. For example, expanded roles for rehabilitation providers are often referred to as Extended Scope Practitioners in the United Kingdom and Advanced Practice therapists in Canada. Given the variation in terminology, these health care providers will be referred to as expanded role providers (ERPs) throughout this paper.

#### Ongoing Management

Health care providers working in expanded clinical roles to provide ongoing management to patients with arthritis while working alongside a specialist, most often a rheumatologist, fulfill a similar role to team care for ongoing management and monitoring over time. This model has most often been used in the outpatient hospital setting for patients with various types of arthritis, most commonly inflammatory arthritis. Patients are typically referred by a primary care physician to a rheumatologist who does the initial assessment of the patient. Appropriate patients are then referred to an ERP, most often a nurse practitioner or clinical nurse specialist, and in some cases, a physiotherapist or occupational therapist, for ongoing monitoring and management over time.

ERPs perform musculoskeletal examinations, do ongoing monitoring, make recommendations regarding changes to medications, and make referrals to other health care providers. For complex disease, patients may require medical intervention necessitating consultation with a rheumatologist, but an ERP may still act as the primary contact for the patient and as a case manager. For more stable disease, patients may be managed by this health care provider, with less frequent follow-up by the rheumatologist. Patient education and self-management are often integral components of the model, including education about medications. It also has the advantage of promoting a focus on psychosocial issues and facilitating continuity of care.

#### Triage

This model typically services patients with musculoskeletal conditions including arthritis. In this model, patients are referred from a primary care physician for consultation with a specially trained ERP or team to conduct an assessment and make decisions about the need for orthopaedic surgeon consultation as well as disease management. The composition of the triage team varies with some teams led by extended scope physiotherapists and other teams consisting of physiotherapists and primary care physicians with special interests in orthopaedics. In some programs, there is a greater emphasis on a multidisciplinary approach with podiatrists and occupational therapists included in the team.

#### Rural Consultation Support

We identified two types of models of care focused on providing local access to specialist care usually in rural and remote communities through traveling health care providers and telemedicine.

The first model, Rural Consultation Support, provides services for patients with different types of arthritis, frequently inflammatory arthritis, living in rural and remote locations, including Aboriginal communities. Patients are referred by a primary care physician or, in some remote communities a nurse, to the specialist who travels to local communities on a regular basis to provide assessment and management of patients in a local clinic. These referrals may be coordinated through a centralized coordinating system. As specialist visits are relatively infrequent and for a defined and short period of time, an important feature is screening of patients by a local health professional to ensure the need for specialist input. The further ongoing monitoring and liaison with the specialist is then often managed by the local health professional. This professional may be a physiotherapist or nurse working in an expanded role or a primary care physician with a special interest in musculoskeletal care.

#### Telemedicine

The second model, Telemedicine, is a means of sharing health information and providing health care services using telecommunications.[45,46] This model has been used to promote access to specialist care. After the patient is referred from the primary care physician to a specialist, telemedicine is used to link the patient to the specialist in a remote location. A key feature of the use of telemedicine in arthritis is the need to examine the joints, which is a hands-on process. A nurse, physiotherapist and/or primary care physician (ideally with experience in joint examination) are present with the patient locally in order to perform the musculoskeletal assessment while the specialist views the process. This is a key difference from other telemedicine for many conditions. Key informants most commonly described using this technology for follow-up of stable patients with inflammatory arthritis, rather than the initial consultation.

### Strengths and Challenges of Models of Care

The strengths of the Specialized Arthritis Program Model identified by key informants were that it provides: 'one stop shopping' or access to a number of service providers in one setting, continuity of care, client-centred care, and access to health care providers with in-depth arthritis skills and knowledge to meet the needs of complex patients. The challenges reported by key informants included: providing access to services for a large geographic base; coordinating multidisciplinary services; and, perceived high cost of using a multidisciplinary team. The challenge of moving away from disease-specific silos and integrating arthritis into a chronic disease model was also identified. Further issues related to ongoing care including the ability of patients to re-access services once discharged, and lack of health care resources in the community to refer patients for further services.

In contrast to the Specialized Arthritis Program model, the main advantages of models using health care providers in expanded clinical roles (Ongoing Management and Triage) related to access to care. Key informants reported these models had potential to rationalize the use of specialist resources and to decrease waiting times for specialist care, by freeing up specialist time. It was also identified that these models promote the use of the most appropriate providers to assess and manage patients by maximizing the use of health professionals' skills and competencies, and facilitate linkages between specialists and other health care providers. Key informants also identified a number of challenges. These include the lack of transferability of the role across settings; lack of recognition and awareness of roles by patients, health care providers, the public and administration; potential lack of acceptance by the public and physicians; lack of structures for billing for consultation services in physician fee-for-service funding models; and lack of communication between primary and secondary care.

Similarly to the Ongoing Management and Triage models, the Rural Consultation Support model and Telemedicine both facilitate timely access to care, including decreased wait times for specialist services. Our key informants also reported they promote linkages and partnerships within communities in different settings, facilitate central coordination of health care, and facilitate communication and coordination amongst health care providers. The challenges that were identified by key informants were shortages of health care providers in communities to provide ongoing care, inadequate knowledge and awareness of arthritis by local health care providers, and lack of public knowledge and awareness of arthritis.

### Improving Arthritis Care Delivery

Key informants were asked to describe their perspective on ideal elements of any interdisciplinary care model for arthritis. They identified a number of key elements for improving and developing arthritis models of care. The common themes that emerged from the interviews are described in Table 2. Key informants identified the need for patients to have the confidence and skills to self manage their condition and health care providers to have the knowledge and skills to diagnose and manage arthritis effectively. Multidisciplinary health care teams with well defined roles and good communication processes were considered a requisite for optimal care although it was generally recognized that access to a full team might not be necessary for all patients. Key informants identified the importance of client-centred, evidenced-based, comprehensive health care that is coordinated across the continuum. Structures at the health system level, such as funding and use of technology to share patient information, were also identified. Linking to the community was considered integral to arthritis care including referrals to community resources and linkages among organizations and community programs. Finally, it was recognized that increasing public awareness and implementing primary prevention strategies is required for broader arthritis management at the population level.

**Table 2:** Key Elements Identified to Improve Arthritis Models of Care

**1. Patient Self-Management**
▪ Patients manage their care through support for self-management
▪ Patient-centred education considering individual patient needs and readiness for information

**2. Provider skills and expertise**

▪ Providers are confident and skilled in musculoskeletal examination and knowledgeable of arthritis care and best practices

**3. Team structure and process**

▪ Multidisciplinary composition of team as needed
▪ Mechanisms for communication/interaction within team including the patient
▪ The team has a clear understanding of the roles in the team
▪ Mutual respect, trust and equality within team
▪ Skills of all providers in the team are maximized

**4. Health Care Delivery**

▪ Timely access
▪ Client-centred approach to care delivery
▪ Coordination of care across the continuum and assistance for patients navigating the system
▪ Comprehensive services including self-management support and non-pharmacologic interventions such as exercise
▪ Evidence-based care
▪ Continuity of care
▪ Develop multiple entry points to system for patients to access care without delay

**5. Health System**

▪ Stable funding
▪ Holistic chronic disease approach
▪ Technology to share patient information across the system
▪ Monitoring systems and research and evaluation
▪ Leadership and local champions
▪ Health human resource planning

**6. Community**

▪ Partnerships between organizations and community programs
▪ Clients are linked to community services at the right stage of the disease process
▪ Community involvement

**7. Public Awareness and Primary Prevention**

▪ Public awareness of arthritis
▪ Primary prevention strategies

## Discussion

Our research highlights the diversity of emerging models of care for arthritis specialist services. Where it differs from other research is in the classification of models of care by key features and focus on comparative strengths of different models of care and the context of which they are best developed. Although the key informants we interviewed described different programs and services, our findings suggest that there are five common types of models of care with characteristics in common. These typologies can be used as a framework that can be complemented by program specific information when describing and discussing models of care. They also may also be used as groupings when examining and comparing the outcomes of models of care. Finally, this research illustrates gaps in health care delivery. In particular, there was a lack of formalized arthritis models of care in community settings with a coordinated flow of patients through the system.

The types of models of care that evolved from our research can be used to characterize programs and services presented in other arthritis research. For example, the specialized arthritis program is most similar to traditional team care which has been studied in both outpatient and inpatient settings.[32,33,43] Since the use of the term 'team care' has been used broadly, our definition of Specialized Arthritis Program is specific to multidisciplinary team care most commonly provided in one location specifically for arthritis. Our research highlighted the differences in models using health care providers in expanded roles with two types of models of care, triage and ongoing management. A number of other studies have examined patient satisfaction and patient outcomes in programs using a nurse practitioner, clinical nurse specialist, physiotherapist practitioner or extended scope physiotherapist.[34,35,39-42] Although telemedicine has been previously evaluated in the literature[45,46], the description of the model in the arthritis context emphasizes the role of other health care providers in the assessment, consultation, and follow-up process. The rural consultation support model has not been well documented or evaluated in the literature.

There are likely to be several factors that influence the development of models of care including setting (urban/rural), type and stage of arthritis, and local health human resources. While this paper examines the key features of the models of care (e.g. team members, care delivery methods, patient population and setting), and begins to examine the context, there is further work needed to identify and explore other factors that affect the choice of models of care. In particular, factors such as the health care environment, population demographics, health human resource availability, and health system organization require further exploration.

In this paper, we focused on models of care that involved specialists and at least one other health care provider. As such, most of the models of care tend to focus on services for individuals with inflammatory arthritis or more advanced osteoarthritis. In addition, most of the models of care presented here tend to provide specialist support to patients for time limited periods or on an episodic basis. All specialist models at some stage imply ongoing need for care in the community. Although the models of care addressed in this paper often referred patients to community services, few address the management of arthritis in primary care. There is also a large population of people with arthritis, particularly osteoarthritis, who manage their disease with support only from primary health care providers, with or without connection to community programs (e.g. for exercise or self-management). Models of service delivery in primary health care that support the management of arthritis is a gap in the health system and there is a need for growth in this area.

There is also opportunity for development in existing models of care. Models with health care providers in expanded roles maximize the roles of all health care providers and free up the time of the specialist for interventions only they can provide. Currently the roles of ERPs focus on triage, ongoing management, and follow-up primarily in secondary care. To achieve further development of ERPs, a collaborative approach to standardize training, provision of professional support for these roles and evaluation is critical. There is opportunity for this role to expand in primary care where these health care providers could be the first point of contact for assessment and management as well as facilitation of appropriate management by primary care physicians, specialists or community services. These uses of ERPs in primary care have not been described in the literature. Other developments in models of care may build on current models such as Specialized Arthritis Programs to 1) facilitate linkages with primary health care and community services to ensure ongoing care in the community, and 2) integrate services across chronic conditions.

Many of the components of arthritis care delivery identified by key informants were similar to elements of the Chronic Care Model (CCM), a conceptual model of chronic disease management developed by Wagner et al.[5,7,8] The model describes essential elements of chronic disease care which are: Self-Management Support, Delivery System re-design, Decision Support, Community, Clinical Information Systems and Health Care Organization. Public awareness and primary prevention were gaps in the CCM that were identified by our key informants and were also integrated in more recent adaptations of the CCM in Canada.[6]

There are a number of limitations to our study. A snowball technique was used to identify key informants for pragmatic reasons. With this methodology, there is the risk that key informants from similar perspectives will be recruited limiting the range of experiences. In addition, it may have been easier to identify key informants from secondary care using this methodology as they are seen as experts in arthritis care.

While we had a large sample with international representation from countries with similar health care systems, the majority of our key informants were Canadian. Even with the diverse representation from across countries (including leaders in the arthritis field who were well-networked to national initiatives), there is the possibility that models exist that are not represented in our typologies. This is a rapidly evolving area. Programs and services are always under development, which may push the boundaries of our definitions of these typologies.

Finally, we recruited a number of key informants from primary care settings but were only able to recruit two primary care physicians. This may reflect the gap in more formalized models of care for arthritis management in primary care. It is recognized as an area for further exploration and development.

## Conclusion

With the aging of the babyboomers and growing prevalence of arthritis, the need for arthritis care is unlikely to abate. Ensuring that patients receive timely, quality care will be an ongoing challenge. At least in the short term, the shortages of health human resources are likely to continue. Our research highlights the different types of models of care that have been developed in an attempt to meet this challenge and points to gaps in the current health care system. The challenge for the future is to develop comprehensive models across the continuum of care for the long-term management of arthritis as a chronic disease by melding the best of these existing models and linking to primary care and the community.

## Competing interests

The authors declare that they have no competing interests.

## Authors' contributions

CM contributed to the conception and design of the study, conducted interviews, participated in analysis and interpretation of data and drafted the manuscript. PV contributed to the study design, conducted interviews and participated in analysis and interpretation of data. EMB was Principal Investigator of the study and made substantial contributions to the conception and study design, interpretation of data and contributed to drafting the manuscript. All authors reviewed and approved the final manuscript.

## Appendix 1. Interview Guide

**1. Briefly describe your position and experience related to arthritis care**.

Sample probes: Role, setting (e.g. community vs. hospital), years of experience (working in arthritis care), education

**2. Please describe your approach to the provision of services or programs for arthritis care [i.e. arthritis model of care]**.

Sample probes:

Who is target population (stage of disease, age)?

What interventions/services are provided/available?

Who delivers the interventions?

Describe the referral processes.

Describe the communication processes.

3. Please describe the strengths of this model of care.

4. In your experience, what are the challenges or barriers in this model of care?

5. Please describe your perspective on ideal elements of any interdisciplinary care model for arthritis (e.g. characteristics, structures and processes).

## Pre-publication history

The pre-publication history for this paper can be accessed here:


